# Variability of phenolic and alkaloid content in different plant parts of *Carissa edulis* Vahl and *Zanthoxylum chalybeum* Engl.

**DOI:** 10.1186/s13104-018-3238-4

**Published:** 2018-02-13

**Authors:** Judith Ssali Nantongo, Juventine Boaz Odoi, Grace Abigaba, Samson Gwali

**Affiliations:** National Forestry Resources Research Institute, P.O Box 1752, Kampala, Uganda

**Keywords:** Phenols, Alkaloids, *C. edulis*, *Z. chalybeum*

## Abstract

**Objective:**

The objective of the study was to investigate the relative abundance and effect of post-harvest treatment on total phenolics (TP) and total alkaloids in the leaves and bark of *Carissa edulis* and *Zanthoxylum chalybeum*, which would give an indication of the suitability of leaves as alternative sources of medicine in these plant species.

**Results:**

Results indicated higher levels of total phenolics than total alkaloids in both of the species under both freezing and air drying conditions. While more alkaloids were found in leaves compared to bark, there was no difference in abundance of phenols between the plant parts of both species. Air drying preserved more TPs than freezing and the opposite was true for alkaloids. For sustainability, leaves are recommended as an alternative source of medicine instead of the preferred root or stem bark. However, the choice of whether to dry or freeze will depend on the specific compound of interest. Assessment of spatial variability of medicinal properties is highly recommended.

## Introduction

Plant secondary metabolites represent a large range of molecules mainly involved in mediating plants with their environment. However, they are also known for their biological activity including analgesic, antimicrobial, stimulant, anti-cancer and even poison depending of the dose used [1–3]. For centuries, they have been effectively utilized for the prevention and treatment of multiple health conditions by almost every known culture [4, 5]. Through indigenous knowledge [4, 6–8], plants and plant parts that confer more efficacy have been identified. However, studies indicate that the nature and quantity of phyto-compounds differs among and within plant species and can be organ dependent within an individual [2, 9]. Quantitative or qualitative medicinal properties are also affected by post-harvesting processes [10].

Air drying is the traditional, low cost technique used for decontamination and long-term preservation [7, 11]. Dried extracts have high stability and are easier to handle, standardize, transport and store. Moreover, dried extracts allow the manufacture of solid dosage forms, like tablets and capsules [10]. However, during the drying process medicines are prone to oxidation, hydrolysis, microbial and other environmental degradation [12]. Freezing, on the other hand is assumed to properly preserve the medicinal compounds. Research has shown that these two post-harvest practices may differentially affect the quantity and quality of chemical and biological properties of medicinal compounds [13–15].

*Carissa edulis* Vahl and *Zanthoxylum chalybeum* Engl. are important medicinal species in Uganda for treatment of tuberculosis and associated diseases [7], sickle cells [16], malaria [17], oral/dental diseases [5] and many other ailments [8]. The bark of the roots or stem is the most important source of medicine [7, 16, 18, 19]. The chemical constituents of these species have been well characterized [20–22] and have shown alkaloids and phenols as the most important active constituents of pharmacological importance [23]. Alkaloids and phenolic compounds possess a wide range of pharmacological properties [24, 25]. Unfortunately, over harvesting of roots and bark severely impacts long term survival of affected species. An immediate intervention is to use leaves although the pertinent question is whether leaves can provide the required concentrations. Available studies have presented mixed results [2, 9]. Therefore, this study compared the phenols and alkaloids between the leaves and root or stem bark of *C. edulis* and *Z. chalybeum* under cold storage and air drying. Understanding the effect of storage may also give insights for quality enhancement, indirectly contributing to reduction of harvesting intensities.

## Main text

### Methods

*Carissa edulis* and *Z. chalybeum* have been selected for study based on their wide medicinal values and long term usage within the local communities of Uganda. Leaf and bark samples were collected from sites where the species naturally occur. *Zanthoxylum chalybeum* samples were collected from Budongo forest (1° 37′–2° 00′N, 31° 22′–31° 46′E) while *C. edulis* samples were collected from an open savannah (55° 14′N, 32° 50′E). 60 samples were collected from 30 individuals of each species. Each sample was subdivided and the fresh subsamples were sent to the National Chemotherapeutics Research Laboratories (NCRL) in Kampala, Uganda and immediately frozen at – 20 °C for 2 weeks before analysis. The other subsamples were air dried at room temperature for 2 weeks at the National Forestry Resources Research Institute (NaFORRI) in Kampala, Uganda. A voucher specimen of each species was collected for confirmation and is deposited at the NaFORRI Herbarium. Permission to collect samples from Budongo Forest was given by the National Forestry Authority.

Total phenolics were determined as garlic acid equivalent (mgGAE/g) using Folin–Ciocalteu reagent standard method [26] with some modifications. 10 ml of 50% methanol were added to 100 mg of well ground material and vortexed for 30 min. Sample extracts were kept at − 20 °C for 10 min and later centrifuged at 300 rpm for 10 min. 0.1 ml of each of collected the sample mixture was diluted with 0.4 ml distilled water, 0.25 ml of 2 N Folin–Ciocalteu reagent and 1.25 ml of 20% sodium carbonate and vortexed for 15 min. The absorbance was measured at 725 nm using a Perkin Elmer Lambda 35 UV/VIS double beam spectrophotometer. Different concentrations of garlic acid (0.001, 0.002, 0.004, 0.008 mg/ml) were prepared in methanol for preparation of a standard curve with equation y = 0.023x − 0.006, R^2^ = 0.988. All determinations were analyzed in triplicate and result expressed in mg gallic acid equivalents per g dry weight. The Folin–Ciocalteu reagent and the gallic acid standard were purchased from Sigma-Aldrich, Inc. (St. Louis, MO, USA).

Alkaloids were estimated gravimetrically. Briefly, 200 ml of 10% acetic acid in ethanol was added to 5 g of the sample. The mixture was covered and allowed to stand for 4 h, then filtered and the extract concentrated to one-quarter of the original volume. Concentrated ammonium hydroxide is added drop wise in order to precipitate the alkaloids. A pre-weighed filter paper was used to filter off the precipitate which was then washed with 1% ammonium hydroxide solution. The filter paper containing the precipitate was dried in an oven at 60 °C for 30 min and then reweighed when cool until a constant weight was obtained and recorded. The weight of the alkaloid which was the difference between the clean and used filter paper was calculated and expressed as g/100 g. All the tests were conducted in triplicates. Means and variances were generated and analyzed using R-studio version 3.4.0 [27]. Graphs were generated in MS Excel.

### Results

**Fig. 1:**
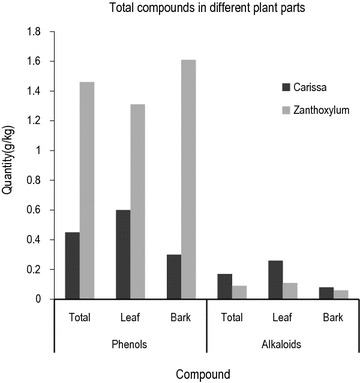
Total alkaloid and phenolic content in leaves and bark of *C. edulis* and *Z. chalybeum*

The two species were different in metabolite concentrations (F_1, 133_ = 1 8.6, p < 0). In both species, the mean quantity of total phenols (9.53 g/kg) was higher than of the alkaloids (1.28 g/kg, 95% CI − 1.13 to 0.52). The phenolic compounds were higher in *Z. chalybeum* (14.58 g/kg) than *C. edulis* (4.47 g/kg) (Fig. 1). The mean of total phenols in the leaves (9.54 g/kg) was not statistically different from that of the bark (9.51 g/kg, 95% CI − 0.39 to 0.28) (Fig. 1). After postharvest processing, the mean of total phenols of the dried material was higher (mean = 17.06 g/kg) than the frozen samples (mean = 1.99 g/kg).

**Fig. 2:**
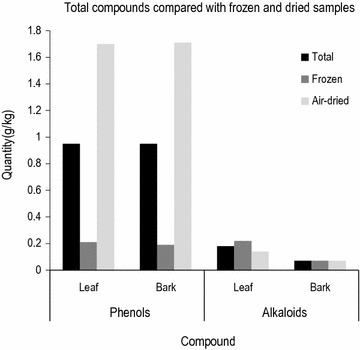
Total alkaloids and phenols in the dried and frozen leaf and bark samples

*Carissa edulis* had more total alkaloids (mean = 1.72 g/kg) than *Z. chalybeum* (mean = 0.84 g/kg) (Fig. 1). In both species, the total alkaloids in the leaves (mean = 1.8261 g/kg) was higher than that of the bark (0.7367 g/kg) (F_1, 70_, t = 4.8 = 25.4). In the frozen samples, the alkaloids were more (Mean = 1.49 g/kg) than the dried samples (mean = 1.07 g//kg) (Fig. 2). There are also highly significant interactions between the species and the chemical compound (F_1, 133_ = 26.3, p < 0), species and storage (F_1, 133_ = 19.2, p < 0), compound and storage (F_1, 133_ = 52.4, p < 0) and between species and plant part (F_1, 133_ = 2.9, p < 0.05).

### Discussion

Phenolics, together with alkaloids, represent some of the main groups of plant secondary metabolites with health benefits [1, 3, 28], rendering them of putative practical interest in pharmacology and related fields. In the study, more total phenols than alkaloids in *C. edulis* and *Z. chalybeum* were detected. Phenolics are the most pronounced secondary metabolites found in [28]. Phenolic compounds are usually related to defense against biotic and abiotic stressors in plants [28–30], although they are also implicated in diverse biological activities such as symbiotic relationships (pollination, nitrogen fixation and seed dispersal) and participating in signalization and structural roles [28, 31]. On the other hand, alkaloids are found in about 20% of the plant species and only in young, actively growing tissues. Consequently their production and abundance are influenced by factors that affect growth of fresh plant tissues, such as light, soil nutrients and moisture, temperature etc. [32]. Generally, in most plants the common order of secondary metabolites with respect to abundance is phenolics > alkaloids > cyanogenic glycosides > tannins > flavonoids and saponins > terpenoids.

The abundance of total phenols was higher in *Z. chalybeum* than *C. edulis* and the opposite was true for alkaloids. Differences in metabolite abundance have been detected among and within species primarily due to genetic factors [30, 33, 34], environmental effects and their interaction [30, 35]. Changing growth conditions, especially nitrogen (N) availability, have been shown to affect phenolic concentrations in plant tissues. Specifically, N deficiency or limitation leads to phenolic accumulation in different plant parts [30, 31]. Comparatively higher levels of constitutive secondary metabolites observed in *Z. chalybeum* may also reflect the level of biotic and abiotic stresses it experiences [35]. These stresses are typical of natural forests where *Z. chalybeum* samples were collected. *C. edulis* was collected from a cattle grazing area, which receives constant N-enrichment from cattle droppings. Also, biotic stresses hence high investment in constitutive phenolics of *C. edulis* is greatly reduced by the long thorns that deter large herbivores and the constant cattle spray that reduces the pest incidences. Optimal defense theory predicts that individuals or tissues that are unlikely to be attacked by pests or pathogens should have low constitutive amounts of defense. The presence of more phenols in *Z. chalybeum* would also be supported by the resource availability hypothesis that predicts more secondary constitutive metabolites in slow-growing plant species [30, 36].

Even though roots and stem bark are exploited more than any other plant parts for medicinal purposes [18, 19], leaves of *Z. chalybeum* and *C. edulis* had equally or higher abundance of compounds. This is probably because leaves are more exposed to stressors than roots and the stem bark [35]. Research has demonstrated organ dependent accumulation of secondary metabolites [37] although no specific trend has been established [21, 38, 39]. Most intra-specific variations have been explained by the carbon/nutrient balance hypothesis that predicts leaves to be more responsive to C:N variations and over a very short time period [40]. Qualitative and quantitative variability due to plant ontogeny, physiological constraints and life-history strategy is also characteristic secondary metabolites [21, 29, 34, 39–41].

The effect of thermal exposure on bioactive compounds has been investigated in a number of plant species and most of these have indicated a degradation of alkaloids on exposure to heat and an enhancement of phenols [15, 18, 42–44]. Enhancement of phenolic compounds has been associated with thermal destruction of cell walls and subcellular compartments that results in increased levels of free phenolic compounds, the formation of novel compounds or the inactivation of deteriorative enzymes such as polyphenol oxidases [15]. Alkaloids on the other hand are not very stable. They undergo degradation or decomposition on exposure to air, light, moisture and heat or chemicals [41].

In summary, this study has shown that *C. edulis* and *Z. chalybeum* are rich in both total alkaloids and total phenols both in the leaves and the root/stem bark. Based on this observation, leaves are recommended as a satisfactory substitute for roots and bark as source of medicine. Leaf harvesting is less destructive for most of the medicinal plants since leaves are abundant, regenerate faster and harvesting doesn’t lead to destruction of major plant tissues. The choice to dry or freeze will depend on the compound of interest. Drying quantitatively affects alkaloids but not the phenols.

### Limitations

Spatial and temporal variations are not catered for.
